# Thoracic Imaging Findings in Systemic Lupus Erythematosus

**DOI:** 10.7759/cureus.107535

**Published:** 2026-04-22

**Authors:** Sankeerth Kendyala, Anu Kapoor, Ramakrishna N

**Affiliations:** 1 Radiodiagnosis, Nizam's Institute of Medical Sciences, Hyderabad, IND

**Keywords:** computed tomography, interstitial lung disease, pulmonary arterial hypertension, systemic lupus erythematosus, thoracic manifestations

## Abstract

Introduction: Systemic lupus erythematosus (SLE) is a multisystem autoimmune disease with frequent thoracic involvement, contributing significantly to morbidity and mortality. Computed tomography (CT) plays a vital role in detecting and characterising thoracic manifestations of SLE. The aim of this study was to evaluate the spectrum of thoracic manifestations of systemic lupus erythematosus using CT.

Methods: This prospective cross-sectional observational study was conducted in the Department of Radiodiagnosis at Nizam’s Institute of Medical Sciences, Hyderabad, over a period of 18 months (November 2022 to April 2024). Sixty adult patients diagnosed with SLE and referred for chest CT were included. High-resolution CT, contrast-enhanced CT, or CT pulmonary angiography was performed based on clinical indication. The primary outcome measured was the spectrum and frequency of thoracic manifestations of SLE identified on CT imaging, along with associated extra-thoracic findings. Descriptive statistical analysis was performed, and results were expressed as frequencies, percentages, and means with standard deviations.

Results: The study population had a mean age of 31.8 ± 9.9 years, with a marked female predominance (n=50, 83.3%). Infection was the most common thoracic diagnosis, identified in 32 patients (53.3%), predominantly bacterial in origin (n=27, 45%). Pulmonary arterial hypertension was observed in 30 patients (50%), followed by pulmonary oedema in 11 patients (18.3%). Interstitial lung disease (ILD) and serositis were each noted in 6 patients (10%), with nonspecific interstitial pneumonia being the most frequent ILD pattern (n=4, 6.6%). Less frequent findings included pulmonary thromboembolism (n=3, 5%), lupus pneumonitis (n=2, 3.3%), and diffuse alveolar haemorrhage (n=2, 3.3%). Splenomegaly was the most common additional finding (n=31, 51.7%).

Conclusion: Thoracic involvement in SLE is diverse, with infections and vascular manifestations being the most frequent. CT imaging plays a crucial role in identifying overlapping thoracic patterns, facilitating accurate diagnosis and guiding appropriate clinical management.

## Introduction

Systemic lupus erythematosus (SLE) is a chronic multisystem autoimmune disease characterised by the production of pathogenic autoantibodies leading to immune complex deposition, complement activation, cytokine release, and subsequent tissue and organ damage. Its pathogenesis involves a complex interplay of genetic susceptibility, immunological dysregulation, hormonal influences, and environmental factors, resulting in a highly heterogeneous clinical presentation [1].

The global prevalence of SLE varies widely, ranging from 13 to 7,713.5 per 100,000 people [2]. In India, the reported point prevalence is approximately 3.2 per 100,000 individuals [3]. Epidemiological studies consistently demonstrate a marked female predominance, with nearly 80%-90% of patients being women of reproductive age, particularly between 15 and 44 years of age [4]. Variations in disease prevalence, severity, and clinical expression across age, gender, and ethnicity suggest the influence of hormonal, genetic, and environmental triggers.

Clinically, SLE presents with a broad spectrum of manifestations, ranging from mild cutaneous and musculoskeletal involvement to severe, life-threatening multisystem disease. The absence of a single pathognomonic clinical feature or laboratory test makes diagnosis challenging, necessitating reliance on a combination of clinical and immunological criteria. Currently, the Systemic Lupus International Collaborating Clinics (SLICC) criteria are widely used for the diagnosis and classification of SLE.

Thoracic involvement is common in SLE, occurring in approximately 50% of patients during the course of the disease and contributing significantly to morbidity and mortality. All compartments of the thorax may be affected, including pleural and pericardial involvement with effusions, pulmonary parenchymal disease such as pneumonitis, interstitial lung disease (ILD), and infections, airway abnormalities, vascular complications including pulmonary arterial hypertension and thromboembolic events, cardiac involvement, and diaphragmatic dysfunction.

The pattern of thoracic involvement is closely related to disease activity. Acute disease flares are associated with potentially fatal manifestations such as diffuse alveolar haemorrhage and acute lupus pneumonitis, whereas chronic disease may lead to interstitial fibrosis and restrictive lung disease [5]. In addition, patients with SLE are at an increased risk of respiratory infections due to intrinsic immune dysfunction and the widespread use of immunosuppressive therapy. Infections account for nearly 60% of secondary thoracic manifestations and are responsible for approximately 30%-50% of SLE-related mortality [6].

Thoracic manifestations in SLE may be primary, directly related to disease activity, or secondary, resulting from infections, drug toxicity, thromboembolic disease, or malignancy. Certain immunosuppressive agents, including methotrexate and biologic therapies such as rituximab, are known to cause drug-induced pneumonitis and ILD. Additionally, SLE is associated with an increased risk of malignancies, particularly pulmonary and lymphoproliferative cancers [7].

Computed tomography (CT), particularly high-resolution CT (HRCT) and CT pulmonary angiography (CTPA), plays a pivotal role in the evaluation of thoracic manifestations in SLE. CT enables early detection of subtle pulmonary abnormalities, accurate assessment of disease extent, and differentiation between overlapping entities such as infection, lupus pneumonitis, organising pneumonia, interstitial lung disease, pulmonary oedema, and pulmonary thromboembolism.

Given the wide spectrum of thoracic involvement and the frequent overlap of imaging features, a pattern-based CT approach is essential for accurate diagnosis and optimal patient management. The present study was therefore undertaken to evaluate the spectrum of thoracic manifestations in SLE using CT and to categorise these findings into distinct radiological diagnoses.

## Materials and methods

This prospective cross-sectional observational study was conducted in the Department of Radiodiagnosis at Nizam’s Institute of Medical Sciences (NIMS), Hyderabad, over a period of 18 months from November 2022 to April 2024. Prior ethical clearance was obtained from the NIMS Institutional Ethics Committee (review letter no. EC/NIMS/3075/2022, dated February 3, 2023), following the committee's review and approval of the protocol titled “Thoracic Imaging Findings in Systemic Lupus Erythematosus and Their Correlation With Serum Biomarkers”.

A convenient sample size of a minimum of 60 participants was determined based on a monthly average of three to four cases matching the criteria during the study period. The study population comprised adult patients diagnosed with systemic lupus erythematosus who presented with thoracic symptoms and abnormal chest CT findings. Patients were excluded from the study if they were pregnant, had an overlap with mixed connective tissue disorders, or were unwilling to provide consent.

All patients included in the study underwent a detailed clinical evaluation followed by image acquisition. CT examinations (HRCT/contrast-enhanced CT (CECT)/CTPA) were performed according to standard protocols using either a 128-slice CT scanner (SOMATOM Definition AS+; Siemens, Germany) or a 16-slice CT scanner (Philips BRILLIANCE™; Philips, Netherlands). The chest CT scans were acquired with the patient lying in a supine position with breath-holding at end-inspiration.

Data were analysed using MS Excel (Microsoft Corporation, Redmond, USA). Descriptive statistics were elaborated in the form of means with standard deviations and medians with interquartile ranges (IQRs) for continuous variables, while frequencies and percentages were calculated for categorical variables. Data were presented graphically wherever appropriate for visualization, utilizing bar charts and pie charts for categorical data.

## Results

The study comprised a total of 60 subjects diagnosed with SLE. The demographic analysis demonstrated a significant female preponderance, with 50 (83.3%) female participants compared to 10 (16.7%) male participants (Table 1).

**Table 1:** Gender distribution of the study population

Gender	No. of patients (%)
Male	10 (16.7)
Female	50 (83.3)
Total	60 (100)

The age of the study population ranged from under 20 to 60 years, with a mean age of 31.8 ± 9.9 years. The majority of participants were young adults, with the 21- to 30-year age group constituting the largest segment (25 patients, 41.6%). This was followed by the 31- to 40-year group (13 patients, 21.6%) and the 41- to 50-year group (11 patients, 18.4%). The extremes of the age spectrum were less represented, with 8 (13.4%) patients aged ≤20 years and only 3 (5%) patients aged 51-60 years (Table 2).

**Table 2:** Age distribution of the study population (mean age, 31.8 ± 9.9 years)

Age group (years)	No. of patients (%)
≤20	8 (13.4)
21–30	25 (41.6)
31–40	13 (21.6)
41–50	11 (18.4)
51–60	3 (5)
Total	60 (100)

All patients underwent chest CT examinations according to standard imaging protocols tailored to the clinical indication. HRCT was performed for the majority of the study population (52 patients), while CECT was conducted for 3 patients, and CTPA was utilized for 5 patients.

**Figure 1:**
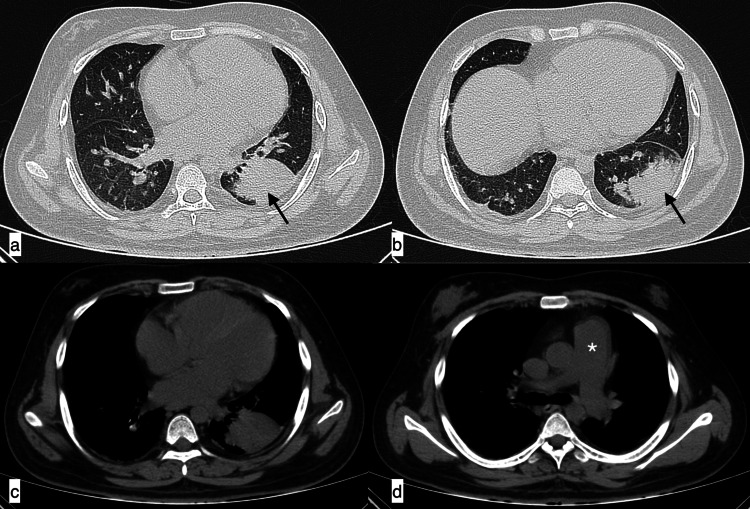
Infective aetiology A 27-year-old male patient with SLE presented with fever, shortness of breath, and cough of seven-day duration. Axial HRCT chest images in the lung window (a, b) revealed a large area of consolidation in the left lower lobe with minimal synpneumonic left pleural effusion (black arrows), suggestive of infective aetiology. Additionally, in the mediastinal window, cardiomegaly (c) and an enlarged pulmonary artery (d, asterisk) were observed. SLE: systemic lupus erythematosus; HRCT: high-resolution CT

**Figure 2:**
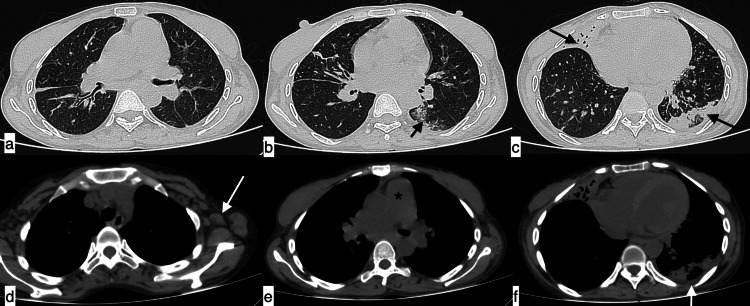
Infective aetiology - community-acquired pneumonia A 31-year-old female patient with SLE presented with complaints of fever, cough, chest pain, and shortness of breath of seven-day duration. Axial HRCT chest images in the lung window (a-c) revealed patchy areas of consolidation (black arrows) in the right middle lobe and basal segment of the left lower lobe with minimal left synpneumonic pleural effusion (f). Additionally, an enlarged pulmonary artery (e, asterisk), cardiomegaly, and axillary lymphadenopathy (d, white arrow) were observed in mediastinal window images. Features were suggestive of infective aetiology, which was confirmed clinically as community-acquired pneumonia. SLE: systemic lupus erythematosus; HRCT: high-resolution CT

**Figure 3:**
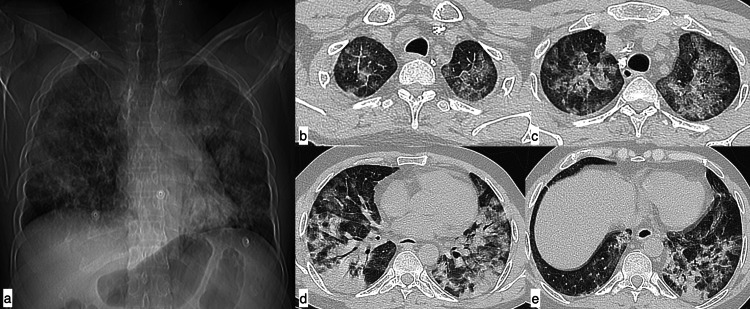
Infective aetiology - polymicrobial infection A 40-year-old male patient with SLE presented with fever, shortness of breath, chest pain, and cough of 11-day duration. The topogram (a) showed patchy consolidations in bilateral lung fields. Axial HRCT chest images in the lung window (b-e) showed interlobular septal thickening with ground-glass opacities with patchy areas of consolidation in bilateral lung fields. Diagnosis of infective aetiology was considered, which was later confirmed as polymicrobial infection (Pneumocystis carinii, bacterial). SLE: systemic lupus erythematosus; HRCT: high-resolution CT

**Figure 4:**
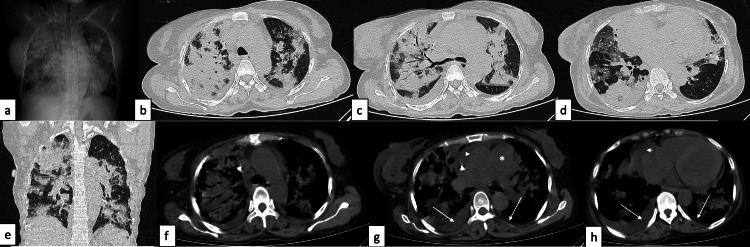
Fungal infection - Aspergillus fumigatus A 45-year-old female patient with SLE presented with complaints of fever, cough, chest pain, and shortness of breath of 22-day duration. (a) The topogram image showed extensive areas of consolidation in bilateral lung fields. Axial (b-d) and coronal (e) HRCT chest images in the lung window confirmed extensive areas of consolidation in both lungs with ground-glass opacities in the right middle lobe. (f-h) Additionally, a dilated main pulmonary artery (g, asterisk), cardiomegaly with mild pericardial effusion (arrowheads), and bilateral mild pleural effusions (white arrows) were observed in the mediastinal window. Features were suggestive of infective aetiology, likely bacterial. Culture from bronchoalveolar lavage confirmed fungal infection (Aspergillus fumigatus). SLE: systemic lupus erythematosus; HRCT: high-resolution CT

**Figure 5:**
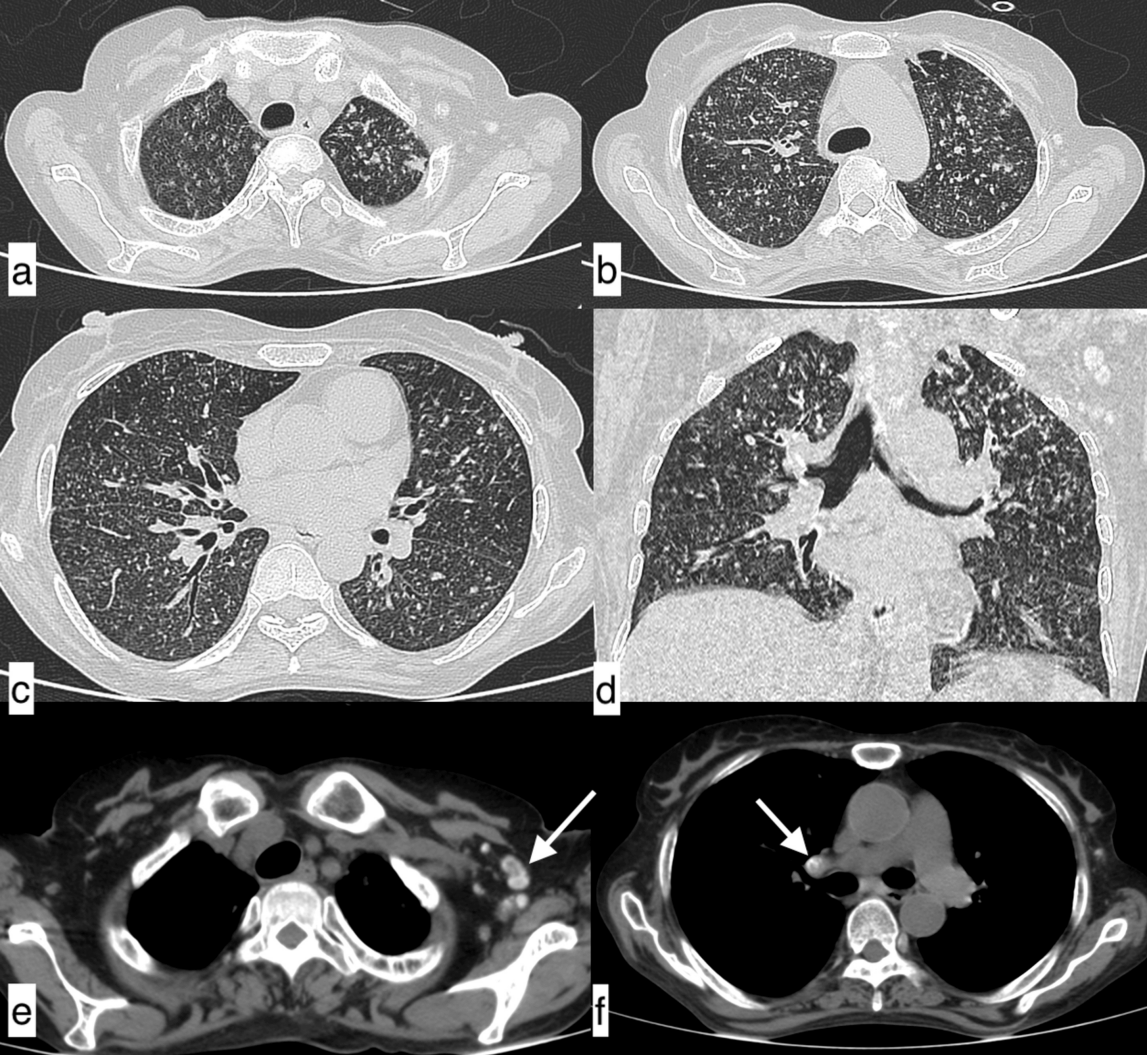
Miliary tuberculosis A 48-year-old female patient with SLE presented with complaints of cough, fever, and shortness of breath of 15-day duration. Axial (a-c) and coronal (d) HRCT chest images in the lung window revealed multiple randomly distributed miliary nodules in both lung fields. Multiple calcified lymph nodes (white arrows) in the left axillary (e) and right hilar (f) regions were observed in the mediastinal window. Findings were suggestive of miliary tuberculosis. SLE: systemic lupus erythematosus; HRCT: high-resolution CT

**Figure 6:**
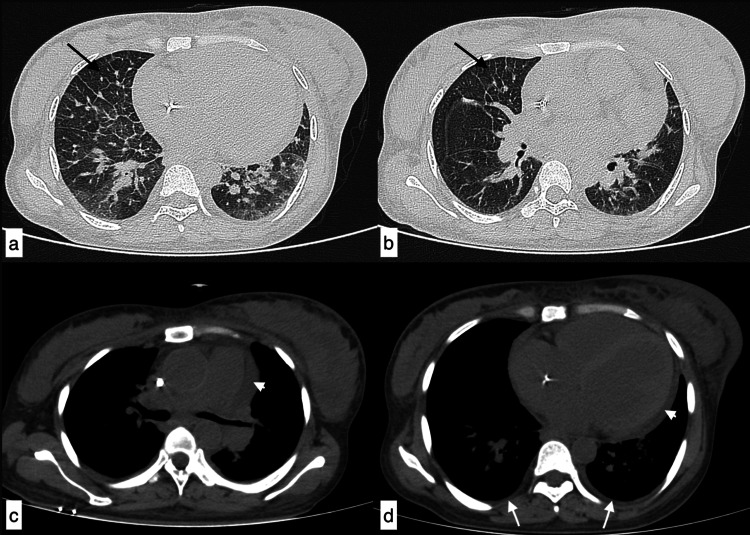
Pulmonary oedema A 25-year-old female patient with SLE presented with shortness of breath and orthopnoea of three-day duration. Axial HRCT chest images in the lung window (a, b) revealed smooth interlobular interstitial septal thickening in both lung fields with diffuse ground-glass opacities in both lung fields, giving a crazy paving appearance (black arrow). Additionally, cardiomegaly with mild pericardial effusion (arrowheads) and minimal bilateral pleural effusions (arrows) were observed in mediastinal window images (c, d). Findings were consistent with pulmonary oedema. SLE: systemic lupus erythematosus; HRCT: high-resolution CT

**Figure 7:**
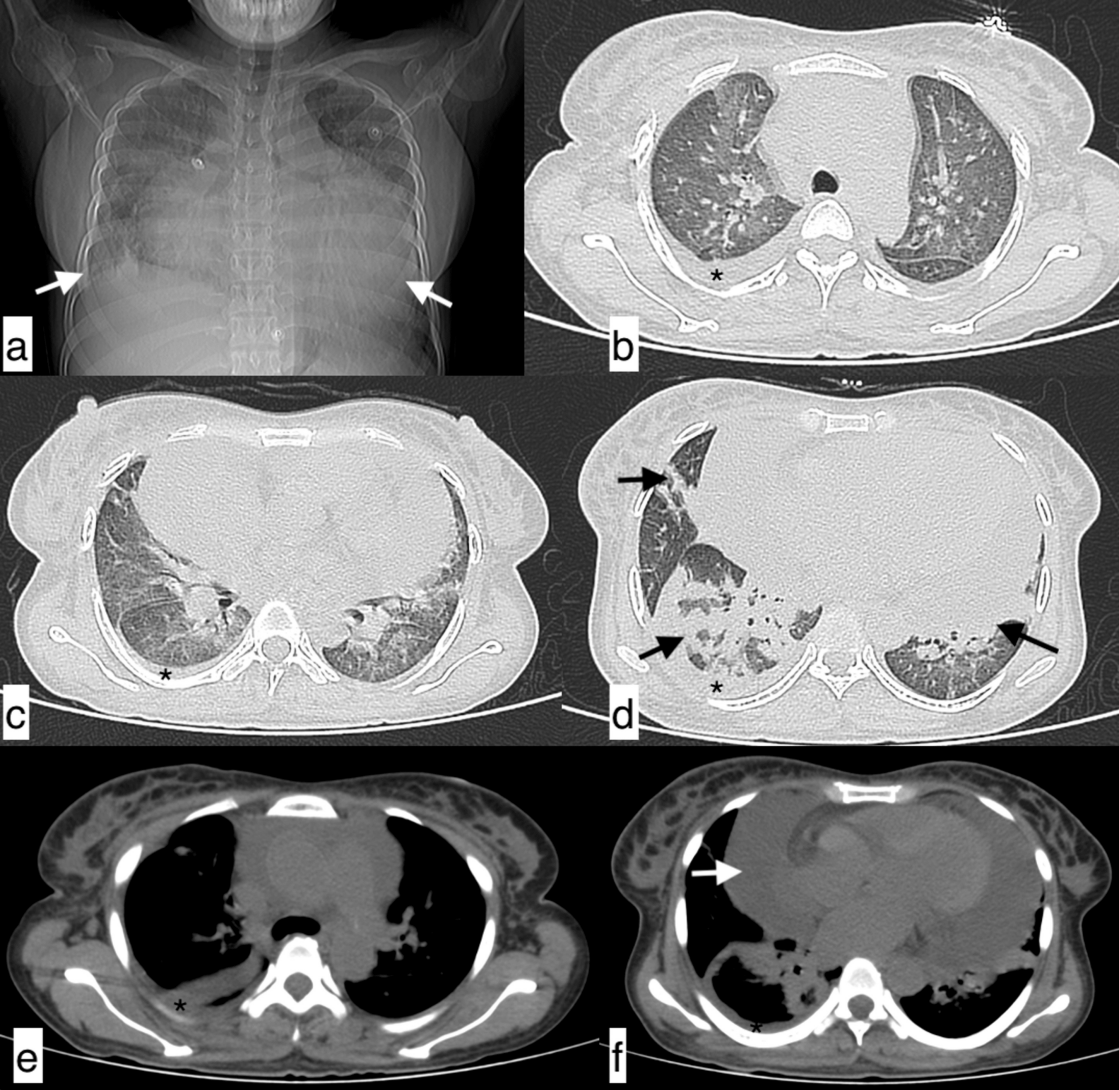
Pulmonary oedema with super-added infection A 25-year-old male patient with SLE presented with complaints of fever, shortness of breath, and cough of nine-day duration. The topogram (a) revealed gross pericardial effusion and blunting of bilateral costophrenic angles (white arrows) with diffuse ground-glass haziness in bilateral lung fields. Axial (b-f) HRCT chest images in the lung window (b-d) revealed diffuse ground-glass attenuation and interlobular septal thickening in bilateral lung fields, with patchy consolidation (black arrows) in the middle lobe, right lower lobe, and left lower lobe. Topogram findings were confirmed as mild right pleural effusion (b-f, asterisk) tracking along the fissure and cardiomegaly with massive pericardial effusion (f, white arrow). Features were suggestive of pulmonary oedema with super-added infection (Pseudomonas aeruginosa). SLE: systemic lupus erythematosus; HRCT: high-resolution CT

Infection was the most common diagnosis, identified in 32 (53.3%) patients (Figures 1, 2). Among the infectious etiologies, bacterial infections were predominant, accounting for 27 (45%) cases. Other infectious causes included polymicrobial (Figure 3) and fungal infections (2 cases each, 3.3%) (Figure 4) and tubercular infection (1 case, 1.67%) (Figure 5). Pulmonary arterial hypertension was also highly prevalent, diagnosed in 30 (50%) patients. Pulmonary oedema was observed in 11 (18.3%) patients (Figures 6, 7).

**Figure 8:**
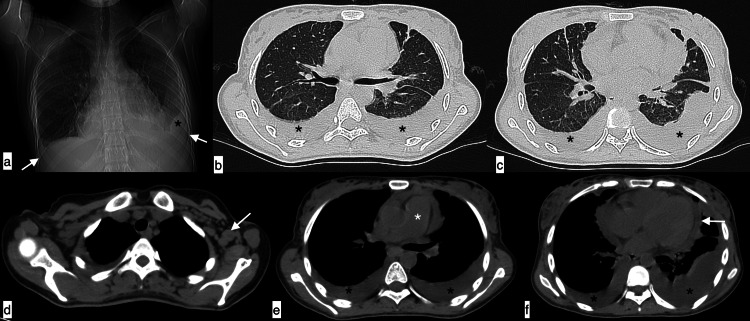
Serositis A 29-year-old female patient with SLE presented with chest pain, fever, and cough of seven-day duration. The topogram (a) revealed blunting of bilateral costophrenic angles (white arrows) with suggested loculated pleural effusion on the left side (black asterisk). Axial chest HRCT images (b-f) confirmed bilateral moderate pleural effusions (black asterisk), pericardial effusion (f, white arrows), cardiomegaly, enlarged pulmonary artery (e, white asterisk), and axillary lymphadenopathy (d, white arrow). Features were suggestive of serositis. SLE: systemic lupus erythematosus; HRCT: high-resolution CT

**Figure 9:**
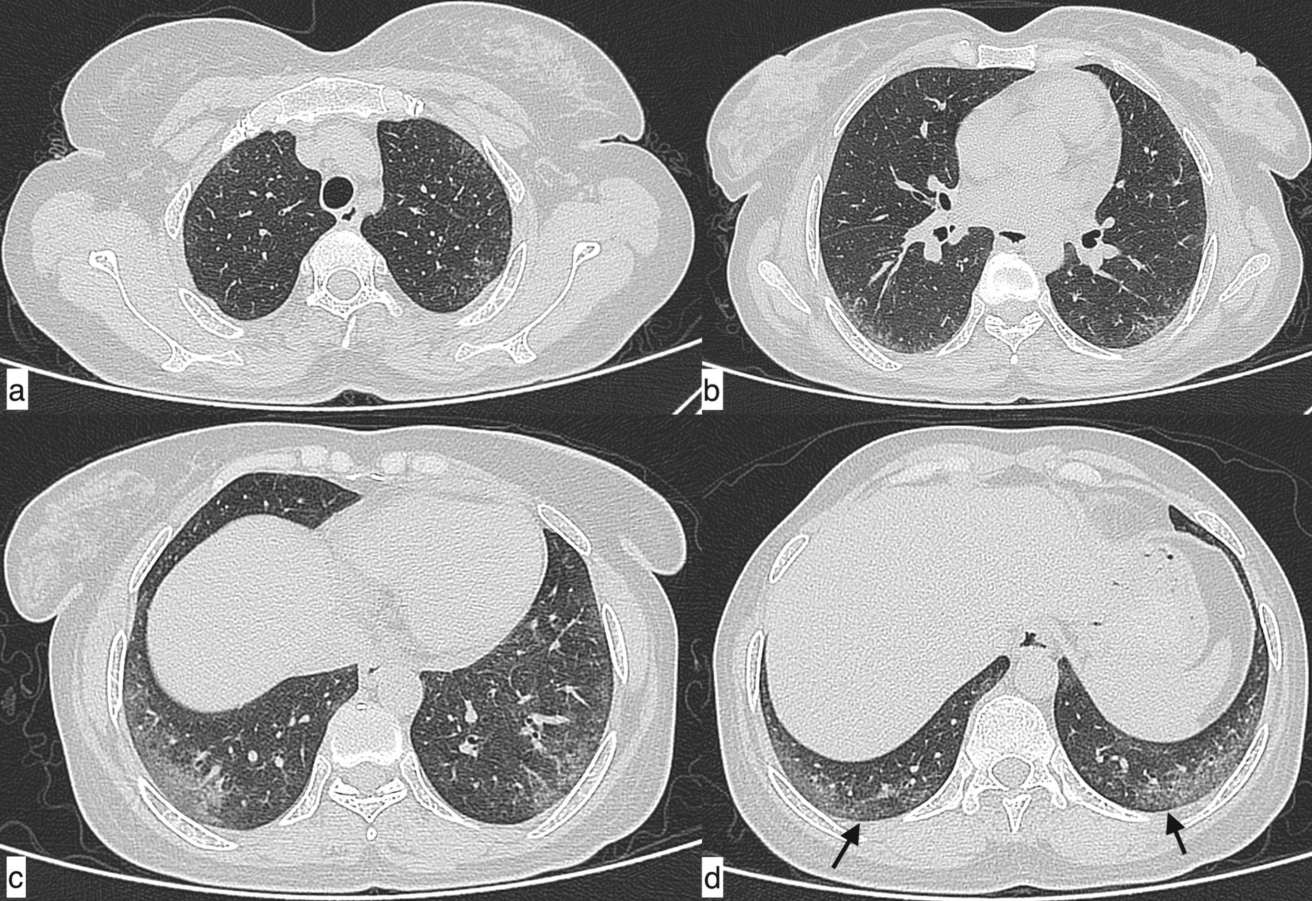
NSIP pattern of ILD A 42-year-old female patient with SLE presented with a four-month history of mild cough and shortness of breath. Axial HRCT chest images in the lung window (a-d) revealed ground-glass opacities with interlobular interstitial septal thickening located peripherally in both lung fields with an apicobasal gradient and subpleural sparing (black arrows), suggestive of the NSIP pattern of ILD. SLE: systemic lupus erythematosus; HRCT: high-resolution CT; NSIP: nonspecific interstitial pneumonia; ILD: interstitial lung disease

**Figure 10:**
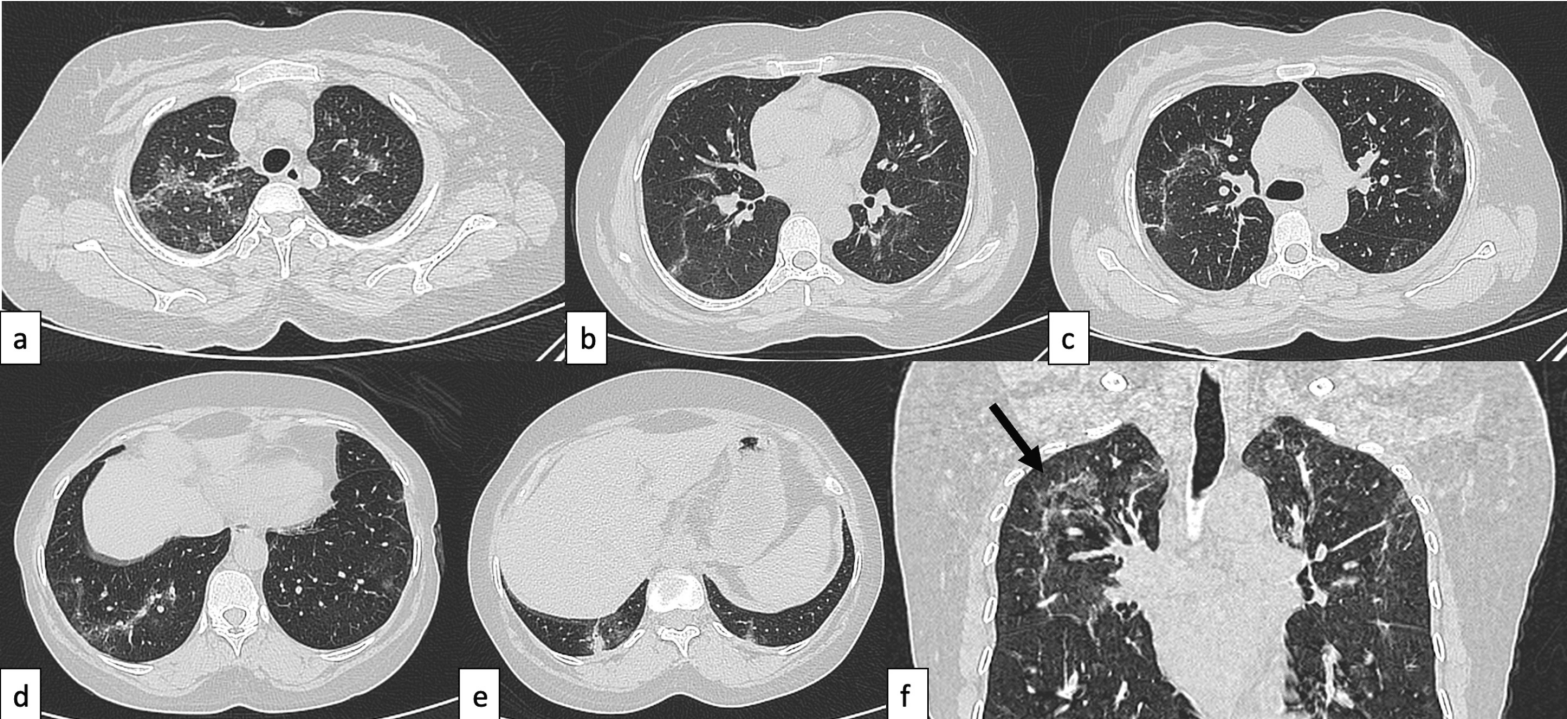
Organising pneumonia A 41-year-old female patient with SLE presented with complaints of shortness of breath, non-productive cough, generalized weakness, and occasional fever of 40-day duration. Axial (a-e) and coronal (f) HRCT chest images in the lung window revealed patchy consolidation and ground-glass opacities in a perilobular pattern, in bilateral lung fields, giving the atoll sign appearance (black arrow). Features were consistent with organising pneumonia. SLE: systemic lupus erythematosus; HRCT: high-resolution CT

**Figure 11:**
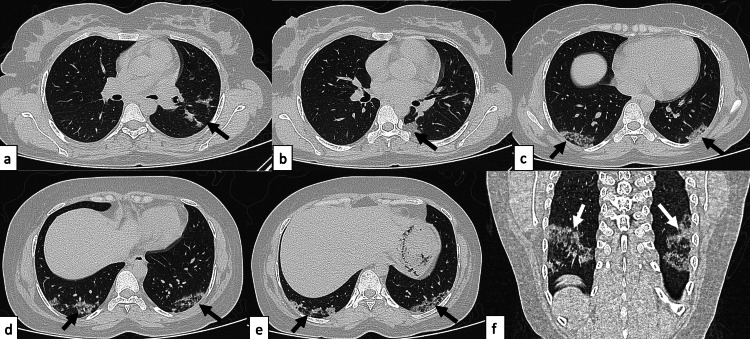
Organising pneumonia A 24-year-old female patient with SLE presented with complaints of dry cough of one-month duration. Axial (a-e) and coronal (f) HRCT chest images in the lung window revealed patchy areas of consolidation predominantly in subpleural locations (black arrows) noted in the bilateral lower lobes with a perilobular pattern of distribution, showing a reverse halo sign (white arrows) suggestive of organising pneumonia. SLE: systemic lupus erythematosus; HRCT: high-resolution CT

**Figure 12:**
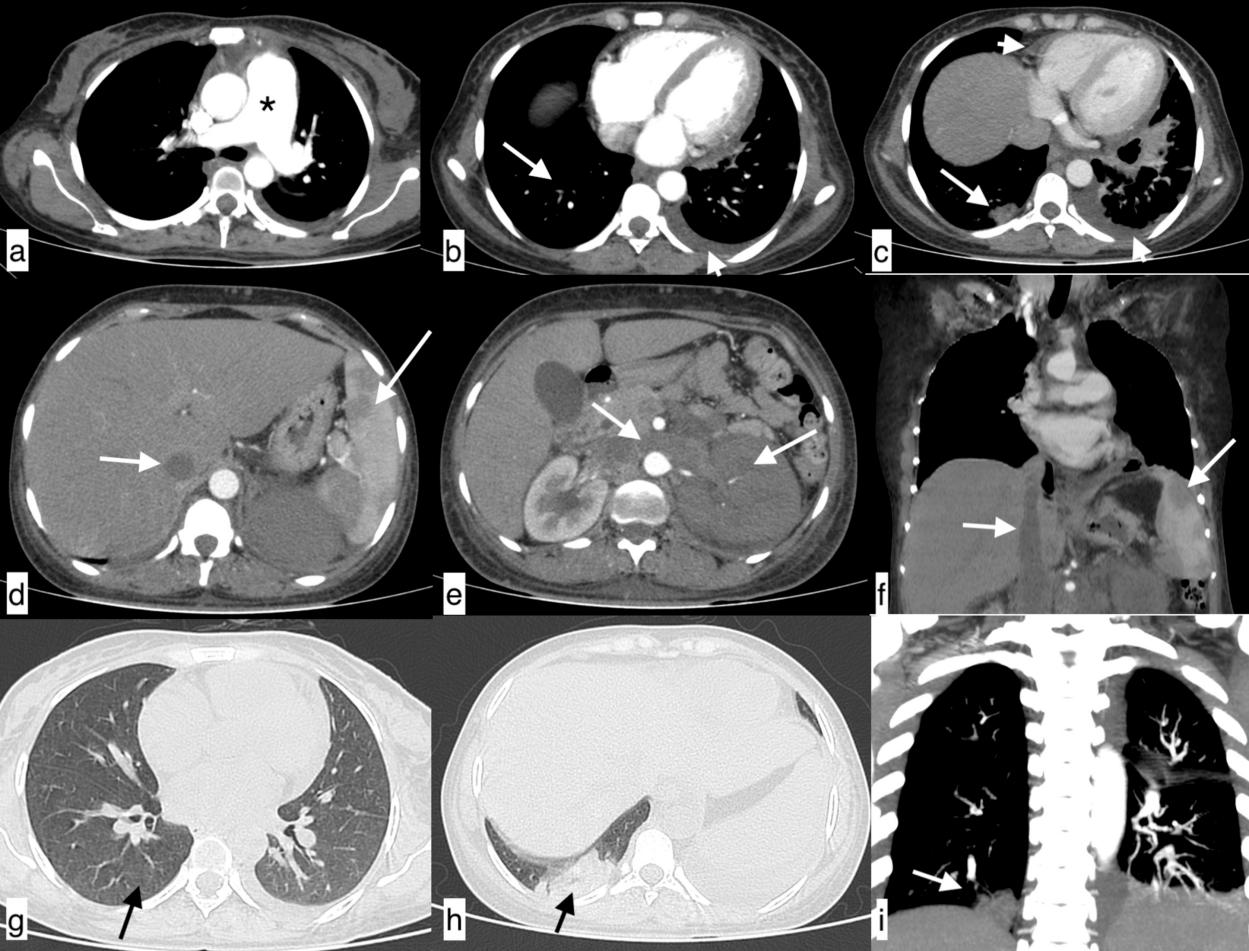
Pulmonary thromboembolism A 28-year-old female patient with SLE presented with complaints of shortness of breath and chest pain of three-day duration. CT pulmonary angiogram revealed a dilated main pulmonary artery (a, asterisk) with hypodense filling defects in the right lower lobar and segmental arteries (b, white arrow), with resultant peripheral subpleural wedge-shaped non-enhancing infarct in the postero-basal segment of the right lower lobe (c, white arrow; g, h, black arrows). Additional findings included thrombosis of the IVC (d, f, white arrows) and left renal vein with a bulky, non-enhancing left kidney (e, white arrows), splenic infarcts (d, f, white arrows), and mild pericardial and left pleural effusion (b, c, arrowheads). Findings were consistent with pulmonary thromboembolism. SLE: systemic lupus erythematosus; HRCT: high-resolution CT; IVC: inferior vena cava

**Figure 13:**
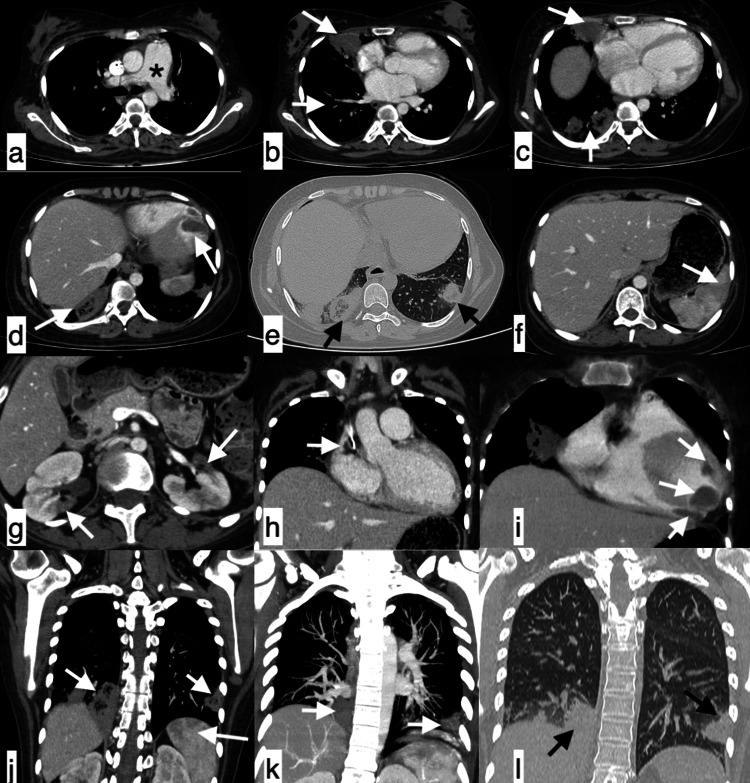
Antiphospholipid syndrome An 18-year-old female patient with antiphospholipid syndrome secondary to SLE presented with shortness of breath and chest pain of two-day duration. CT pulmonary angiogram revealed a dilated main pulmonary artery (a, asterisk) with thrombosis of subsegmental vessels (b, white arrow), with resultant infarcts seen as areas of wedge-shaped consolidations with a broad base toward the pleura, along with a few air bronchograms within the medial segment of the right middle lobe. Areas of ground-glass opacities with surrounding consolidation extending to the subpleural location showed a reverse halo sign in the bilateral lower lobes (b-e, j-l, white and black arrows). Additional findings included a large eccentric filling defect in the distal SVC around the central line (h, white arrow), a non-enhancing thrombus in the left ventricle and right ventricle near the apex (i, white arrows), cardiomegaly with left ventricle and left atrium dilation (c), splenic infarcts (f, j, white arrows), and renal infarcts (g, white arrows). SLE: systemic lupus erythematosus; SVC: superior vena cava

**Figure 14:**
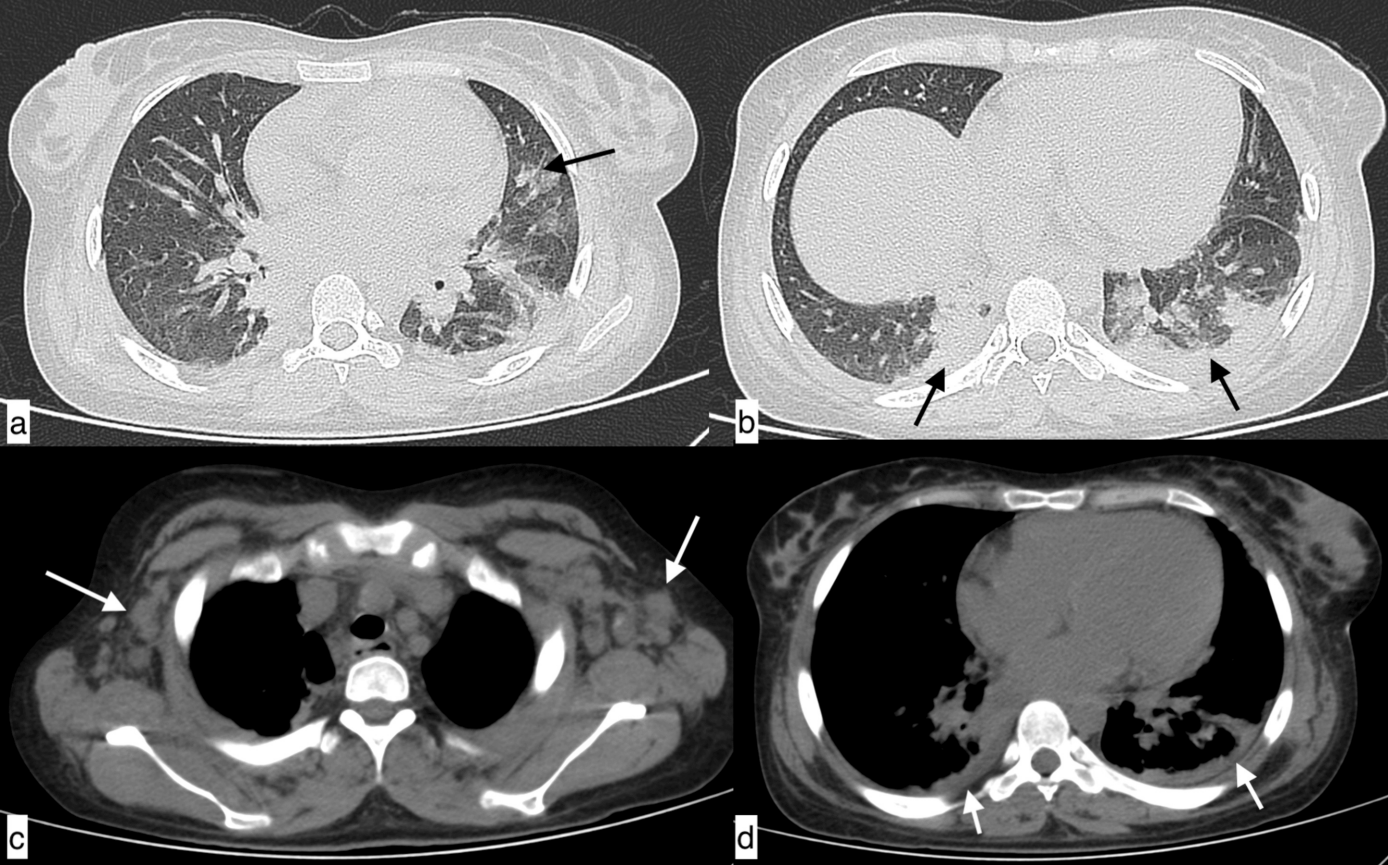
Lupus pneumonitis An 18-year-old female patient with SLE presented with fever, shortness of breath, skin rash, joint pains, and myalgias of five-day duration. Axial HRCT chest images in the lung window revealed patchy areas of consolidation (black arrows) in the lingular segment of the left upper lobe (a) and basal segments of bilateral lower lobes (b). Mediastinal window images showed enlarged bilateral axillary lymph nodes (c) and cardiomegaly with bilateral minimal pleural effusions (white arrows) (d). Imaging differentials included infection and lupus pneumonitis. The final diagnosis was lupus pneumonitis. SLE: systemic lupus erythematosus; HRCT: high-resolution CT

**Figure 15:**
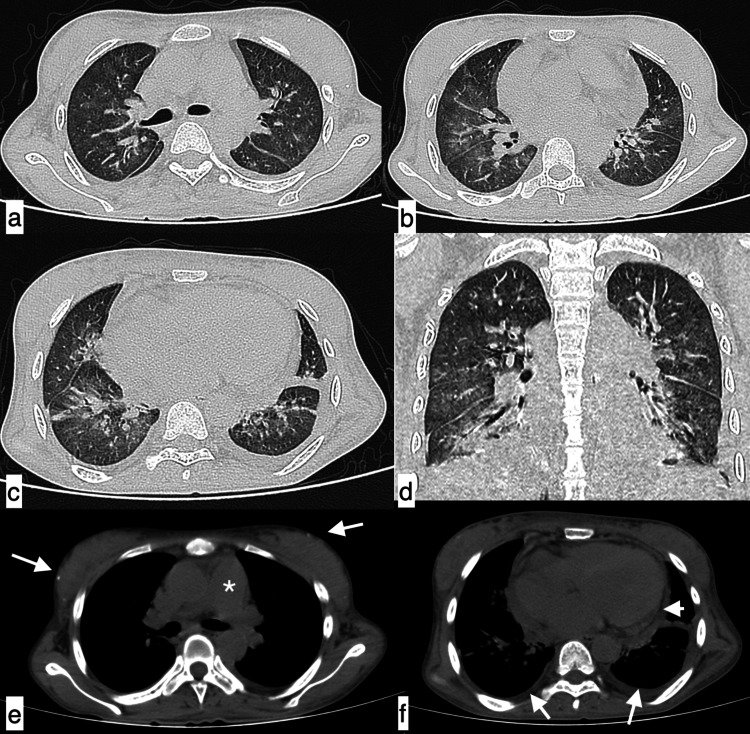
Diffuse alveolar haemorrhage A 27-year-old female patient with SLE presented with complaints of shortness of breath, fever, chest pain, and cough of four-day duration. Axial (a-c) and coronal (d) HRCT chest images in the lung window revealed symmetrical ground-glass opacities in both central and peripheral lung fields. Additionally, in the mediastinal window (e, f), an enlarged pulmonary artery (asterisk), mild pericardial (f, arrowhead) and bilateral pleural effusions (f, arrows), and cardiomegaly were also noted. Incidentally, vascular calcifications were observed in both breasts (e, arrows). Findings were consistent with diffuse alveolar haemorrhage. SLE: systemic lupus erythematosus; HRCT: high-resolution CT

**Figure 16:**
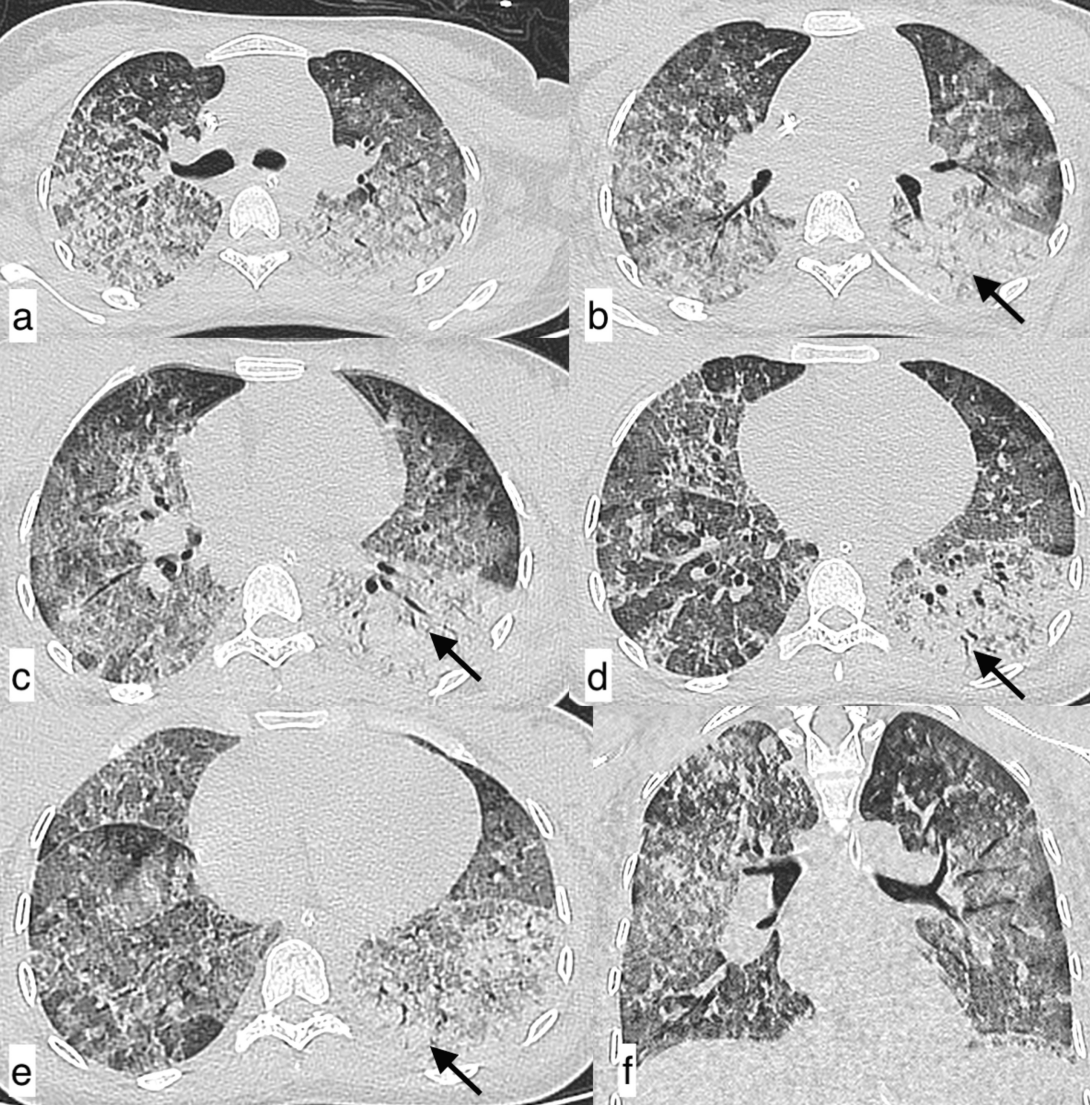
Diffuse alveolar haemorrhage with coexistent infection A 26-year-old female patient with SLE presented with haemoptysis, fever, and cough of two-day duration. Axial (a-e) and coronal (f) HRCT chest images in the lung window revealed extensive areas of ground-glass attenuation with associated interlobular septal thickening in both lung fields, with a large area of consolidation (black arrow) in the left lower lobe; imaging diagnosis of diffuse alveolar haemorrhage with coexistent infection was made, which was confirmed clinically. SLE: systemic lupus erythematosus; HRCT: high-resolution CT

ILD and serositis (Figure 8) were each noted in 6 (10%) patients. Within the ILD subset, nonspecific interstitial pneumonia (NSIP) was the most common pattern (4 cases, 6.6%) (Figure 9), followed by organising pneumonia (2 cases, 3.3%) (Figures 10, 11). Sequelae of old infection were identified in 5 (8.3%) cases. Less frequent diagnoses included pulmonary thromboembolism (3 cases, 5%) (Figures 12, 13), lupus pneumonitis (2 cases, 3.3%) (Figure 14), and diffuse alveolar haemorrhage (2 cases, 3.3%) (Figures 15, 16). No cases of shrinking lung syndrome or neoplastic conditions were observed in the study population (Table 3).

**Table 3:** Final diagnosis in the study population

Final diagnosis	Frequency (N=60)
Infection	32
Bacterial	27
Polymicrobial	2
Fungal	2
Tubercular	1
Pulmonary arterial hypertension	30
Pulmonary oedema	11
Interstitial lung disease	6
Nonspecific interstitial pneumonia	4
Organising pneumonia	2
Serositis	6
Sequelae of old infection	5
Pulmonary thromboembolism	3
Lupus pneumonitis	2
Diffuse alveolar haemorrhage	2
Shrinking lung syndrome	0
Neoplastic	0

Beyond the primary thoracic diagnoses, a variety of additional findings affecting different organ systems were recorded. Splenomegaly was the most frequent additional finding, present in 31 (51.67%) patients. This was followed by vascular calcifications in 14 (23.33%) patients and a patulous oesophagus in 13 (21.67%) patients. Calcified lymph nodes were observed in 10 (16.67%) cases.

**Figure 17:**
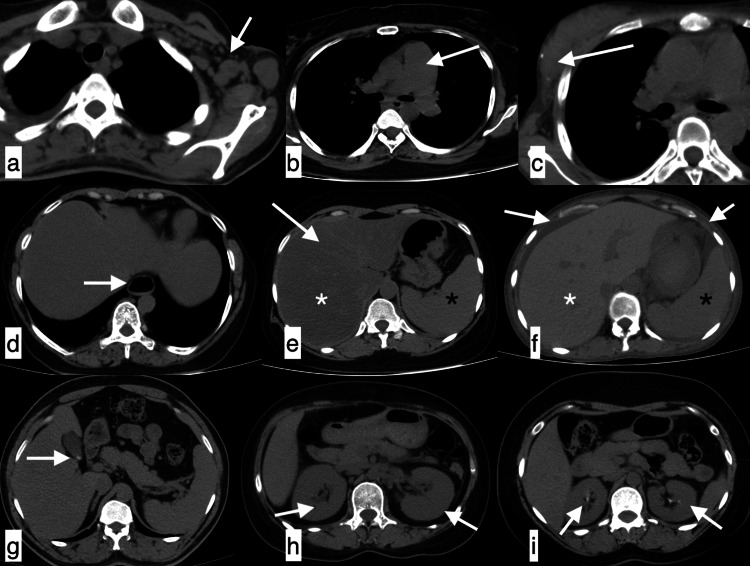
Additional findings A few of the additional findings included axillary lymphadenopathy (a), enlarged pulmonary artery (b), vascular calcifications in breast (c), patulous oesophagus (d), fatty liver with hepatomegaly (white asterisk) and splenomegaly (black asterisk) (e), ascites and anasarca with hepatomegaly (white asterisk) and splenomegaly (black asterisk) (f), cholelithiasis (g), bulky kidneys (h) and renal calculi (i).

Other notable findings included bulky kidneys with perinephric fat stranding (9 patients, 15%), axillary lymphadenopathy (8 patients, 13.33%), and both anasarca and fatty liver (6 patients each, 10%). Hepatomegaly was found in 5 (8.33%) patients. Ascites and oedematous bowel thickening were each observed in 4 (6.67%) patients, while splenic infarcts and renal calculi were seen in 3 (5%) patients each. Cholelithiasis was present in 2 (3.33%) patients. Rare findings, each affecting a single patient (1.67%), included autoimmune hepatitis, increased liver attenuation, dilated ascending aorta, intraventricular thrombus, renal infarcts, renal cortical cysts, renal vein thrombosis, pancreatitis, and inferior vena cava (IVC) thrombus (Table 4, Figure 17).

**Table 4:** Additional findings

Additional findings	No. of positive patients (%)
Splenomegaly	31 (51.67%)
Vascular calcifications	14 (23.33%)
Patulous oesophagus	13 (21.67%)
Calcified lymph nodes	10 (16.67%)
Bulky kidneys with perinephric fat stranding	9 (15%)
Axillary lymphadenopathy	8 (13.33%)
Anasarca	6 (10%)
Fatty liver	6 (10%)
Hepatomegaly	5 (8.33%)
Ascites	4 (6.67%)
Oedematous bowel thickening	4 (6.67%)
Splenic infarcts	3 (5%)
Renal calculi	3 (5%)
Cholelithiasis	2 (3.33%)
Autoimmune hepatitis	1 (1.67%)
Increased attenuation of the liver	1 (1.67%)
Dilated ascending aorta	1 (1.67%)
Intraventricular thrombus	1 (1.67%)
Renal infarcts	1 (1.67%)
Renal cortical cysts	1 (1.67%)
Renal vein thrombosis	1 (1.67%)
Pancreatitis	1 (1.67%)
Inferior vena cava thrombus	1 (1.67%)

## Discussion

Our study evaluated thoracic and associated systemic manifestations in 60 patients with SLE using computed tomography. The findings highlight a predominance of young female patients and demonstrate a wide spectrum of pulmonary and extra-pulmonary involvement, underscoring the multisystemic nature of the disease.

In the present cohort, females constituted the majority of patients (50, 83.3%), with males accounting for only 10 cases (16.7%), yielding a female-to-male ratio of approximately 5:1. This pronounced female preponderance is in keeping with the established epidemiology of SLE. Similar female predominance has been reported by Alamoudi and Attar (86%) [8], Afify et al. (92%) [9], and Narayanaswamy et al. (92%) [10]. Alhammadi et al. [11] reported an even higher proportion of females (95.1%), while Enomoto et al. [12] documented a comparatively lower, yet still predominant, female representation (76.4%). These consistent observations across studies reinforce the role of hormonal, genetic, and immunological factors in the pathogenesis of SLE.

The mean age of patients in this cohort was 31.8 ± 9.9 years, with the majority of patients clustered in the younger age groups. The highest proportion of patients belonged to the 21- to 30-year age group (25, 41.6%), followed by the 31- to 40-year group (13, 21.6%) and the 41- to 50-year group (11, 18.4%). Patients aged ≤20 years constituted 8 cases (13.4%), while only 3 patients (5%) were in the 51- to 60-year age group. This age distribution closely mirrors that reported by Alamoudi and Attar [8], who observed a mean age of 32 ± 13.2 years, and Hamed et al. [13], who reported a mean age of 30 ± 9 years. Narayanaswamy et al. [10] also noted that a substantial proportion of patients were in their third and fourth decades, comparable to the 63.2% of patients aged 21-40 years in the present study.

In contrast, Enomoto et al. [12] reported a higher median age of 54 years, while Li et al. [14] documented a mean age of 44.13 ± 12.17 years. Alhammadi et al. [11] similarly reported an older age distribution, with 41.7% of patients aged 41 years or above. These differences in age distribution may reflect variations in referral patterns, disease duration, geographic factors, and healthcare access across study populations.

Infection emerged as the most common thoracic diagnosis, identified in 32 patients (53.3%). Among these, bacterial infections predominated, accounting for 27 cases (45%), highlighting the increased susceptibility of SLE patients to bacterial pathogens due to immune dysregulation and immunosuppressive therapy. Polymicrobial and fungal infections were each observed in 2 patients (3.3%), while tubercular infection was noted in 1 patient (1.67%), emphasizing the need for tuberculosis screening in endemic regions.

Pulmonary arterial hypertension was diagnosed in 30 patients (50%), indicating a substantial disease burden. It is a known cause of significant morbidity and mortality in SLE, and its high prevalence in the current analysis reinforces the importance of routine screening and early therapeutic intervention.

Pulmonary oedema was observed in 11 patients (18.3%), often reflecting underlying cardiac or renal involvement, both of which are common in SLE. ILD and serositis were each identified in 6 patients (10%). Within the ILD subgroup, NSIP was the predominant pattern (4 patients, 6.6%), followed by organising pneumonia (2 patients, 3.3%). These findings are consistent with previous reports describing NSIP as the most frequent ILD pattern in SLE.

Sequelae of old infections were identified in 5 patients (8.3%), emphasizing the importance of differentiating residual changes from active disease. Less common but clinically significant findings included pulmonary thromboembolism in 3 patients (5%), lupus pneumonitis in 2 patients (3.3%), and diffuse alveolar haemorrhage in 2 patients (3.3%). No cases of shrinking lung syndrome or neoplastic involvement were detected in the present cohort.

Among the extra-pulmonary findings, splenomegaly was the most frequently observed, present in 31 patients (51.67%). This incidence is higher than that reported by Zhang et al. (42.86%) [15] and substantially exceeds the 12% reported by Chowdhary et al. [16]. The higher frequency in the present study may be related to greater disease severity or the presence of systemic complications such as autoimmune haemolytic anaemia, portal hypertension, recurrent infections, or hepatic involvement.

Vascular calcifications were noted in 14 patients (23.33%), while a patulous oesophagus was identified in 13 patients (21.67%). The prevalence of a patulous oesophagus in the present study closely parallels the findings of Sabri et al. [17], who reported oesophageal abnormalities in 25% of SLE patients, highlighting the importance of gastrointestinal involvement in SLE.

Calcified lymph nodes were observed in 10 patients (16.67%), while axillary lymphadenopathy was noted in 8 patients (13.33%). This frequency aligns with the prevalence of lupus lymphadenopathy reported by Cervera et al. [18], who documented rates ranging from 12% to 15% in later stages of the disease. Recognition of lymphadenopathy is crucial, as it necessitates exclusion of infectious, inflammatory, drug-induced, and neoplastic causes.

Renal and abdominal findings were also common. Bulky kidneys with perinephric fat stranding were observed in 9 patients (15%), while anasarca and fatty liver were each identified in 6 patients (10%). The prevalence of fatty liver in the present study lies between that reported by Suzuki et al. (4.6%) [19] and Huang et al. (41%) [20], suggesting the influence of metabolic and demographic factors.

Hepatomegaly was observed in 5 patients (8.33%), while ascites and oedematous bowel thickening were each present in 4 patients (6.67%). Splenic infarcts and renal calculi were noted in 3 patients each (5%). Cholelithiasis was identified in 2 patients (3.33%), a frequency higher than that reported by Suzuki et al. (1.6%) [19] but considerably lower than that reported by Zhang et al. (23.81%) [15].

Several rare findings included autoimmune hepatitis, increased liver attenuation, dilated ascending aorta, intraventricular thrombus, renal infarcts, renal cortical cysts, renal vein thrombosis, pancreatitis, and inferior vena cava thrombosis, each observed in 1 patient (1.67%). These findings further emphasize the diverse systemic involvement seen in SLE.

Differentiating lupus pneumonitis (2 patients, 3.3%), infectious pneumonia (32 patients, 53.3%), and organising pneumonia (2 patients, 3.3%) remains challenging due to overlapping clinical and radiological features. Lupus pneumonitis typically presents with acute respiratory symptoms and bilateral ground-glass opacities, often sparing the peripheral lung zones. Infectious pneumonias demonstrate variable patterns depending on the causative organism, while organising pneumonia commonly shows bilateral peripheral consolidation and ground-glass opacities. Given these overlaps, accurate diagnosis requires correlation of imaging findings with clinical presentation, laboratory markers, microbiological results, and response to therapy. A multidisciplinary approach is therefore essential to guide appropriate management and improve patient outcomes.

The present study has certain limitations. Firstly, the sample size was relatively small, which may limit the generalizability of the findings to the broader population of patients with SLE. A larger sample size could provide more robust statistical power and allow for stronger correlations between imaging findings and clinical outcomes. Future multicentric studies with larger cohorts are warranted to validate and expand upon the observations of this study.

## Conclusions

The present study evaluated the spectrum of thoracic manifestations in systemic lupus erythematosus using computed tomography in a cohort of 60 patients, predominantly young females. Infections, particularly bacterial, emerged as the most common thoracic abnormality, followed by pulmonary arterial hypertension. Pulmonary oedema, interstitial lung disease, and serositis were less frequent but clinically significant findings. Nonspecific interstitial pneumonia was the predominant ILD pattern observed. In addition to thoracic manifestations, several extra-pulmonary findings such as splenomegaly, vascular calcifications, patulous oesophagus, lymphadenopathy, and renal and hepatic abnormalities were commonly detected, reflecting the multisystemic nature of SLE.

Computed tomography proved invaluable in detecting subtle and coexisting thoracic abnormalities, many of which demonstrate overlapping imaging features that can pose diagnostic challenges. Correlation of CT findings with clinical and laboratory parameters is therefore essential for accurate diagnosis. A pattern-based CT approach aids in differentiating disease-related manifestations from secondary complications such as infection or thromboembolism, ultimately contributing to improved patient management and outcomes.
